# The role of Human leukocyte antigen class I on patient survival in Gastrointestinal cancers: a systematic review and meta- analysis

**DOI:** 10.1038/s41598-020-57582-x

**Published:** 2020-01-20

**Authors:** Hadis Najafimehr, Nastaran Hajizadeh, Ehsan Nazemalhosseini-Mojarad, Mohamad Amin Pourhoseingholi, Meghdad Abdollahpour-Alitappeh, Sara Ashtari, Mohammad Reza Zali

**Affiliations:** 1 Basic and Molecular Epidemiology of Gastrointestinal Disorders Research Center, Research Institute for Gastroenterology and Liver Diseases, Shahid Beheshti University of Medical Sciences, Tehran, Iran; 2 Gastroenterology and Liver Diseases Research Center, Research Institute for Gastroenterology and Liver Diseases, Shahid Beheshti University of Medical Sciences, Tehran, Iran; 3 Larestan University of Medical Sciences, Larestan, Iran

**Keywords:** Gastroenterology, Gastrointestinal cancer

## Abstract

The prognostic role of Human leukocyte antigen class I (HLA- I) in gastrointestinal cancers has been remained controversial. We performed a meta-analysis to determine the role of classical HLA-I in predicting survival of patients. In addition, the relationship between HLA- I and some clinicopathological factors was evaluated. Published studies investigated HLA-I expression effect on gastrointestinal cancers were evaluated to determine association between HLA- I and overall survival (OS) and recurrence-free survival (RFS) in patients. The used effect sizes were hazard ratio (HR) and Odds ratio (OR) with 95% confidence interval (CI). A total of ten studies included 1307 patients were analyzed. The pooled results revealed that HLA- I overexpression was positively related to OS (HR: 0.72; 95% CI: 0.53–0.96) and demonstrated little association for RFS (HR: 0.70; 95% CI: 0.46–1.08). HLA-I overexpression is negative associated with poorer differentiation of tumor (OR: 0.53; 95% CI (0.43–0.81) and also higher stages of cancer (OR: 0.29; 95% CI (0.13–0.64). HLA- I overexpression was related to a better prognosis on OS and probably had little impact on RFS.

## Introduction

Recent advances in knowledge on the biology of cancer cells have demonstrated that the adaptive immune system might play a critical role in controlling tumor progress and elimination of metastasis cells especially in solid tumors. However some tumor cells may have an ability to escape from the immune system^1^. In recent years, for managing tumor growth with the aid of antitumor T cells or antibodies responses screening, immunotherapy has been more attended^2–7^. Human leukocyte antigens class I (HLA- I) which are expressed on the tumor cell’s surfaces, are applicable in the surveillance of the T cell immune responses^8^. HLA- I molecules are critical for the presentation of antigen peptides derived from tumor cells to cytotoxic T lymphocytes (CTLs). HLA- I expression have a key role in the tumor cell recognition by CTLs^9^ and determining expression of this antigen helps to predict risk of progression and recurrence of cancers^10^.

To investigate classical HLA- I (HLA- A, HLA- B, HLA- C) expression function on cancerous patients survival, several studies had been performed^11–15^. In some studies loss HLA- I expression has been associated with good prognosis^16,17^ and the others had reported opposite result and loss expression significantly was associated with worse prognosis^18^. Some studies reported high expression was associated with better prognosis while, others did not report any significant association^19,20^. These results are inconsistent and the prognostic role of classical HLA-I remain controversial. To address this gap, the authors performed a systematic review and meta- analysis to assess the prognostic value of classical HLA- I (A, B, C) expression on survival of gastrointestinal cancer patients.

## Materials and Methods

### Search strategies

The authors searched many studies and literatures which were published in English on the association between HLA- I expression and gastrointestinal cancers survival up to 2018. The searches were done in the PubMed, Scopus, Embase and Web of Science data bases and the search terms were as follows: “Human leukocyte antigen class I”, “HLA class one”, “HLA- I”, “expression”, “survival analysis”, “prognostic”, “prognosis” and “Gastrointestinal cancer”. In addition, the references reported in all relevant studies were evaluated for completing searches and it has been avoided the studies with the same results in several publications.

### Inclusion and exclusion criteria

The inclusion criteria were as follows: (1) the clinical research on classical HLA- I expression in the gastrointestinal cancers. (2) the survival analysis with overall survival (OS) and or recurrence free survival (RFS) as outcome. (3) the patients without any limitation on age or sex. (4) the studies reported hazard ratio (HR) and 95% confidence interval (CI) or those could be estimated from other information in the paper. The exclusion criteria were as follows: (1) the studies which had not enough information on survival analysis and HR. (2) studies which had reported impact of HLA expression combined with other clinicopathological factors on survival time. Case reports and review articles were also excluded from the study.

### Data extraction and quality assessment

All studies were checked by authors to select eligible studies regarding Newcastle- Ottawa guideline^21^. The studies with high and moderate quality considered as eligible and enrolled in our research then others were excluded. This guideline caused the authors to be certain any disagreement does not remain. Information from eligible studies were included the first author name, year of publication, country, median follow up time, patients mean age, sample size, name of cancer, treatment, tumor differentiation, stage, number of metastasis patients and result of survival analysis.

### Statistical analysis

HR and 95% CI were extracted from studies which had directly reported them and if there was only Kaplan Miere (KM) curve, these latter were calculated by corresponding’s guideline^22^. Pooled HR was obtained though combining the HR and 95% CI from all eligible studies based on HR meta- analysis guideline^23^. The pooled HRs were obtained for both of OS and RFS modes. An observed HR > 1 implied a worse survival for the patients with positive or overexpression of HLA- I. In addition to HRs, pooled odds ratios (ORs) were also obtained in order to assessing relation between HLA- I expression level and potentially important factors. Heterogeneity among the result of the studies was evaluated by I^2^ criteria. An I^2^ > 50% with P- value < 0.05 implied that the heterogeneity existed. In this condition, the random effect model was used rather than the fixed model in order to report pooled estimate. For publication bias assessing, The Egger’s and Begg’s tests were used. For adjusting the publication bias, a trim and fill analysis was performed and for checking robustness of the pooled estimate results, sensitivity analysis was done. All analyses were calculated by Stata 14 software and P- value < 0.05 was considered as statistically significant.

## Result

### Literature search and characteristics of the eligible studies

**Figure 1:**
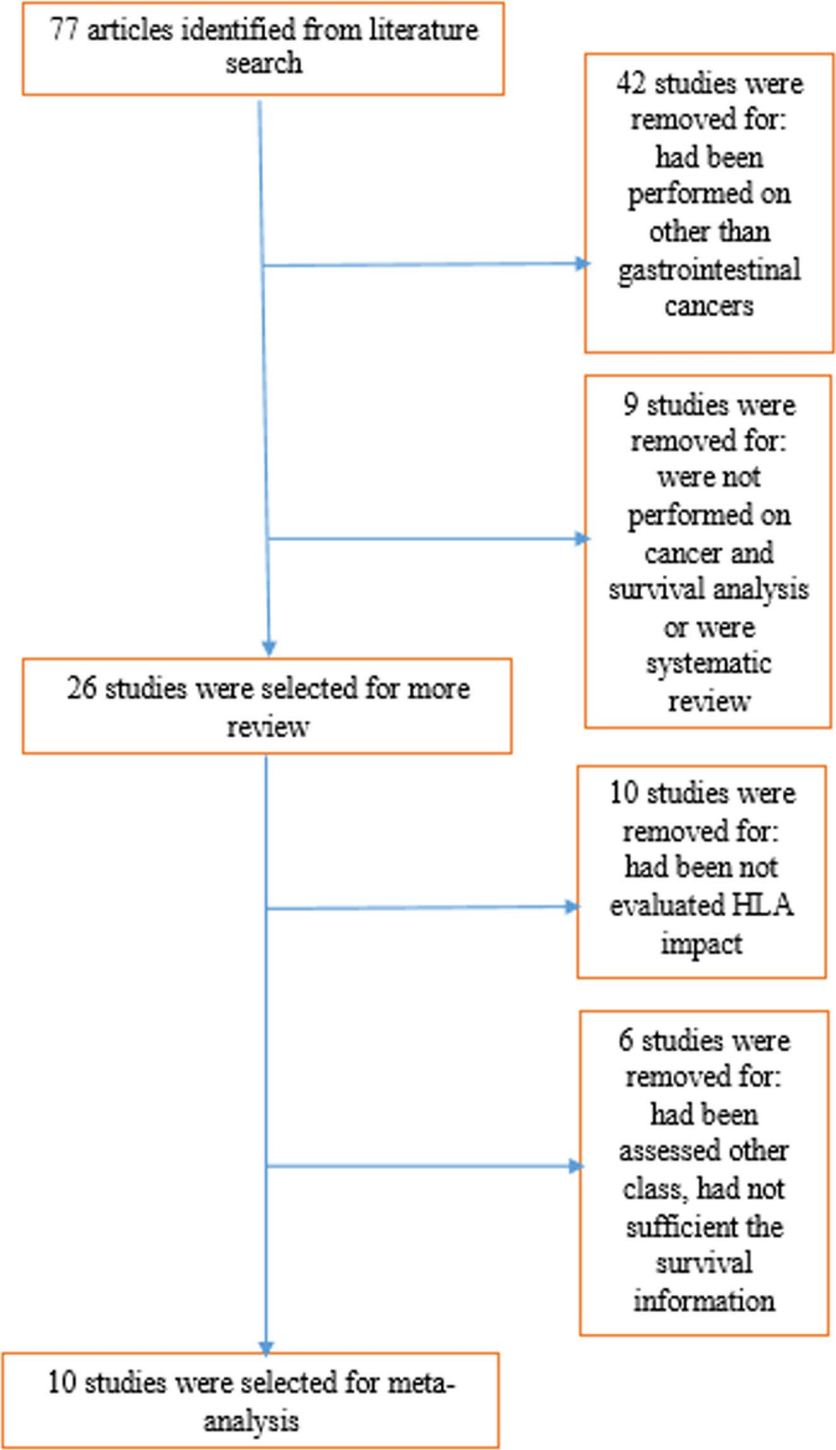
Flowchart of study selection process.

During searching processes, 77 articles were found which were close to research purpose. From them 42 study had not performed on gastrointestinal cancers and were excluded. Nineteen studies had not met inclusion criteria, three studies were on other class of HLA (include G or E)^24–26^ and in the three studies the information about survival analysis were not extractable^10,19,27^ and all mentioned were excluded. Finally 10 studies were include in the meta- analysis^13,28–36^. The flow chart of the literature search is as Fig. 1.

The eligible studies were included 1308 patients and most of patients were over 60 years old. Most studies had been performed in the Asian countries. The main characteristics of selected studies were summarized in Table 1. In these ten cohort studies female and male with stages I, II, III, IV from gastrointestinal cancers were evaluated to assess correlation between survival time and HLA and also other clinicopathological factors. Immunohistochemical analysis were included formalin-fixed and paraffin-embedded tissue blocks. HLA- I expression status including tumor staining in the studies, were according to the international immunogenetics guidelines^37^. Survival analysis with the aid of Cox model in some studies was included OS (outcome had been considered as death) and in the others was included RFS (outcome had been considered as recurrence or progression) and there were some studies with both of them at the same time.

**Table 1:** Main characteristics of 10 studies included in the meta- analysis.

Study	Year	Region	Sample size	Follow- up time (median and range)	Age (mean and range)	Cancer	Predominant treatment	Stage (I/II/III/IV)	Tumor differentiation (well/moderate/poor)	No. of distal metastasis	Survival analysis	Result
Qifeng [28]	2011	China	87	NR	≥ 60: n = 54 < 60: n = 33	Esophageal squamous cell carcinoma	NR	I,II: n = 33; III,IV: n = 54	32/19/36	NR	OS	Worse (positive VS negative)
Ito [29]	2016	Japan	90	NR	62.7(38–82)	Esophageal Squamous Cell Carcinoma	radical surgery without preoperative therapy	NR	36/46/8	47	OS	NS (high VS low)
											RFS	NS (high VS low)
Reimers [30]	2014	Netherlands	495	NR	64.5	Rectal	non-preoperative radiotherapy	134/136/193/32	25/358/110	NR	OS	NS (expression VS loss, downregulation VS loss)
											RFS	good (expression VS loss); NS (downregulation VS loss)
Mizukami [31]	2008	Japan	70	(49–168) m	64.9	Oesophageal squamous cell carcinoma	operated	14/36/24/0	15/38/17	41	OS	Worse (weak + absent VS strong)
TANAKA [32]	2012	Japan	95	95.6 (5.1–183.7) m	63 (46–86)	Advanced esophageal	radical esophagectomy	I,II: n = 54; III,IV: n = 41	NR	0	OS	All stage: worse; I/II: NS; III/IV: worse (downregulation VS up)
Zhang [33]	2012	China	105	14 (4–40) m	≤ 59: n = 50 > 59: n = 55	Esophageal squamous cell carcinoma	Radiotherapy or chemotherapy	I/II: n = 27; III/IV: n = 78	Well + moderate: n = 62; Poor: n = 43	NR	OS	Good (strong VS weak)
Study	Year	Region	Sample size	Follow- up time (median and range)	Age (mean and range)	Cancer	Predominant treatment	Stage (I/II/III/IV)	Tumor differentiation (well/moderate/poor)	No. of distal metastasis	Survival analysis	Result
Umemoto[34]	2014	Japan	80	2427 d	62.8	Hepatocellular carcinoma	Hepatectomy	I,II: n = 21; II,IV: n = 15	Well/moderate: n = 20; poor: n = 16	NR	OS	NS (high VS low)
Imai [35]	2017	Japan	36	NR	68.2	Pancreatic cancer	pancreatic resection	I,II: n = 24; III,IV: n = 12	Well: n = 25; moderate and poor: n = 11	NR	OS	Good (high VS low)
											RFS	Good (high VS low)
Moller [13]	1991	Germany	152	48 (45–65) m	66	Colorectal carcinoma1	Surgery	52/48/52/0	17/112/23	NR	RFS	Worse (normal VS reduced)
Iwayama [36]	2015	Japan	97	54 m	(31–83)	Colorectal cancer	Chemotherapy	II: n = 97	Well/moderate: n = 88; poor: n = 9	NR	RFS	Worse (negative VS positive)

### HLA- I expression and OS in gastrointestinal cancers

**Figure 2:**
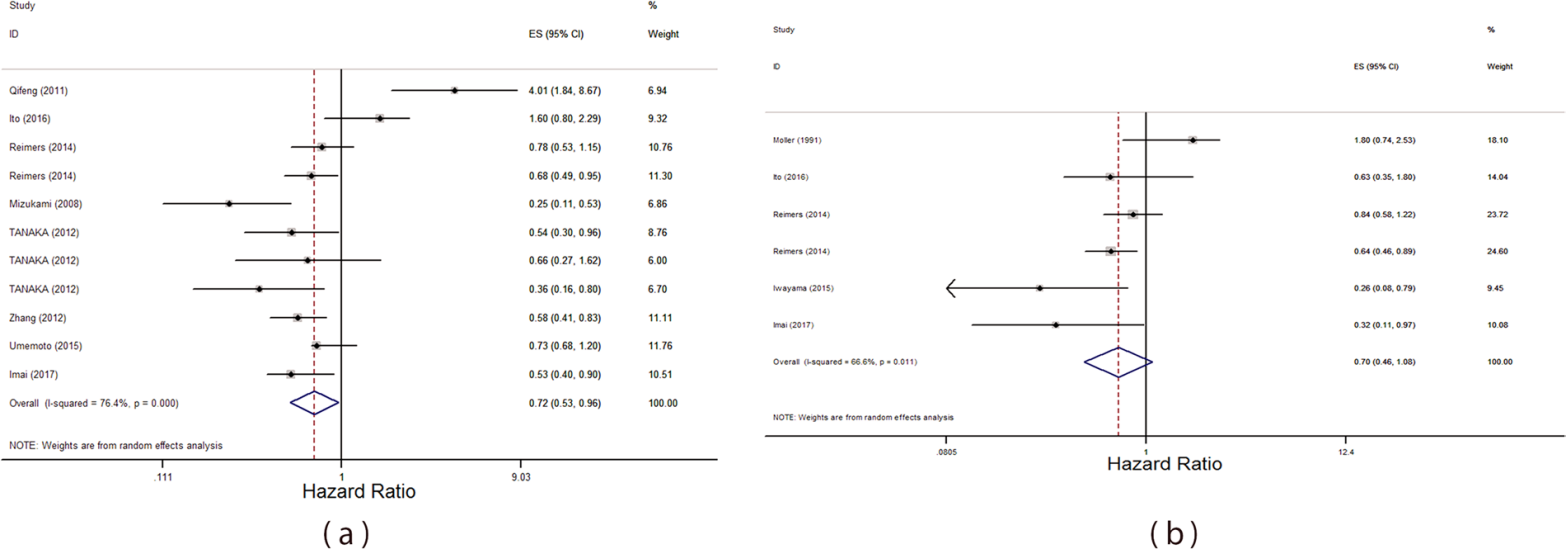
Forrest plot of pooled hazard ratio on the association between HLA- I overexpression and OS (**a**) and RFS (**b**) in gastrointestinal cancers.

There were 8 studies performing follow up which had reported HLA expression and OS for patients^28–35^. Using fixed model, the pooled HR was 0.70; 95% CI (0.62, 0.81), P = 0.03 with I^2^ = 0.76%, P = 0.00. Since the heterogeneity existed, the random effect model was used and the pooled HR was obtained 0.72; 95% CI: 0.53, 0.96, P = 0.03 with I^2^ = 76.4%, P = 0.00. HR demonstrated significant relationship between overexpression and OS increasing. The result is presented in Fig. 2

### HLA- I expression and RFS in gastrointestinal cancers

**Figure 3:**
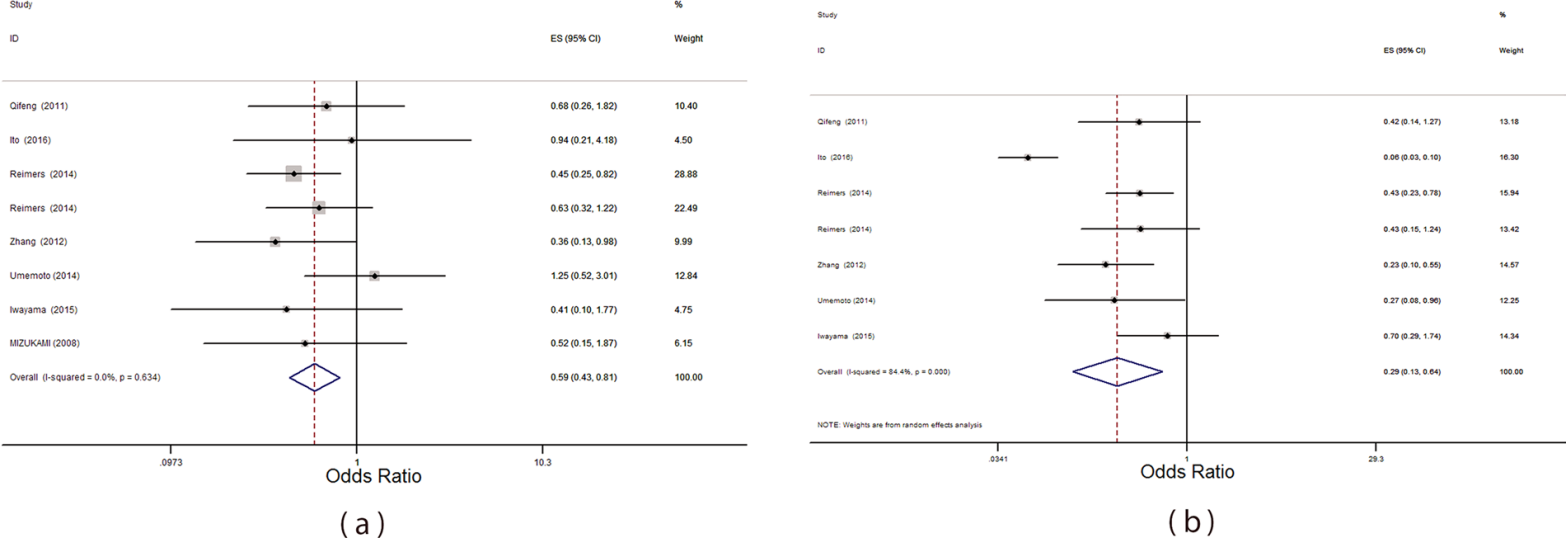
Forrest plot of pooled odds ratio on the association between HLA- I overexpression and tumor differentiation (**a**) and stage (**b**).

There were 5 studies performing follow up on RFS and HLA-I expression^13,29,30,35,36^. The pooled HR using fixed model was 0.75; 95% CI: 0.60, 0.92, P = 0.02 with I2 = 66.9, P = 0.01. The pooled HR by random effect model was 0.70; 95% CI: 0.46, 1.08, P = 0.11 with I^2^ = 66.6%, P = 0.01. The pooled HR demonstrated poor relationship with RFS (Fig. 3).

Because of the heterogeneity remained yet, the subgroup analysis was performed to assess its resources.

### Subgroup meta- analysis

#### Pooled HR by cancer type

Studies were included Esophageal cancer^28,29,31–33^, Colorectal cancer^13,30,36^, Hepatocellular carcinoma^34^ and Pancreatic cancer^35^. The pooled HR for each cancer are presented in Table 2.

**Table 2:** Subgroup Meta- analysis of hazard ratio for HLA- I expression.

Survival analysis	Subgroup	N. of study (N. of ES^*^)	Random effect pooled HR (95% CI)	P	I^2^	P
Overall survival	Cancer					
	Esophageal	5 (7)	0.74 (0.40,1.36)	0.34	85.00%	0.00
	Colorectal	1 (2)	0.72 (0.56,0.93)	0.01	0.00%	0.61
	Pancreatic	1 (1)	0.73 (0.55,0.97)	0.003		
	hepatocellular carcinoma	1 (1)	0.53 (0.35,0.80)	0.03		
	Region					
	Asian	7 (9)	0.71 (0.48,1.06)	0.09	80.9%	0.00
	Non- Asian	1 (2)	0.72 (0.56,0.93)	0.01	0.00%	0.61
	Treatment					
	Surgery	6 (9)	0.65 (0.50,0.84)	0.001	63%	0.006
	Non-surgical	1 (1)	0.58 (0.41,0.83)			
Recurrence- free survival	Cancer					
	Esophageal	1 (1)	0.63 (0.28,1.43)	0.27		
	Colorectal	3 (4)	0.79 (0.47,1.33)	0.38	75.6%	0.006
	Pancreatic	1 (1)	0.32 (0.11,0.95)	0.04		
	Region					
	Asian	3 (3)	0.42 (0.24,0.74)	0.003	0.00%	0.39
	Non- Asian	2 (3)	0.93 (0.56,1.54)	0.78	76.7	0.01
	Treatment					
	Surgery	4 (5)	0.78 (0.52,1.19)	0.26	0.65%	0.02
	Non-surgical	1 (1)	0.26 (0.08,0.81)	0.02		

^*^Effect Size;

#### Pooled HR by region

The eligible studies had been done in the countries of China, Japan, Netherlands and Germany. We performed meta- analysis in two Asian^28,29,31–36^ and Non- Asian^13,30^ groups. The result is presented in Table 2.

#### Pooled HR by treatment

Radiotherapy, chemotherapy or resection were the patient treatments. We performed meta- analysis for predominant treatment which was included surgery (member or tumor removing)^13,29–32,34,35^ and non- surgery (radiotherapy or chemotherapy)^33,36^ as shown in Table 2.

### HLA- I expression and tumor differentiation

There were 7 studies providing data on relation between expression of HLA and tumor differentiation^28–31,33,34,36^. The pooled OR revealed that overexpression had negative association with poorer differentiation (OR: 0.53; 95% CI (0.43, 0.81) and HLA-I overexpression tend to be occurred in the lower level (well and moderate) of differentiation, P = 0.001 with I^2^ = 0.00, P = 0.63). The Forrest plot of these relationship is shown as Fig. 3.

### HLA- I expression and cancer stage

The number of studies on stage and HLA expression was 6^28–30,33,34,36^. The pooled OR revealed that overexpression in stages III, IV was less than stages I, II (OR: 0.29; 95% CI (0.13, 0.64), P = 0.97 with I^2^ = 84.4, P = 0.00) (Fig. 3).

### Publication bias

The Begg’s and Egger’s tests were performed to evaluate publication bias. There was publication bias in OS among studies results (Egger’s P = 0.09, Begg’s P = 0.21). For RFS also publication bias existed (Egger’s P = 0.77, Begg’s P = 0.71). In order to adjust publication bias, the Trim and Fill analysis was used. It was estimated that 2 studies concerning OS were unpublished but for studies concerning on RFS any unpublished study was not found. According to the result of fill meta- analysis for OS and RFS the pooled HRs were 0.52; 95% CI (0.33, 0.72) and 0.72; 95% CI (0.37, 1.07), respectively.

### Sensitivity analysis

The sensitivity analysis was performed with the aid of removing the studies with different results in each of the OS and DFS approach and the pooled HRs were re- estimated. The results showed that the pooled estimates had not a big change.

## Discussion

Prognostic factors were demonstrated to be useful for determination of therapeutic strategies and follow-up examinations in cancer treatment^38^. HLA class I molecules play key roles in the anti-cancer immune system, particularly as a cancer antigen-presenting molecule for cytotoxic T lymphocytes (CTLs)^39^. CTLs have ability to recognize antigenic peptides presented on the cancer cell surface by HLA class I molecules and kill target cancer cells. Nonetheless, cancer cells have ability to escape from the immune system through several ways, including downregulation of HLA class I molecules, secretion of immunosuppressive cytokines, and infiltration of immunosuppressive cells^40^. Such observations demonstrated that the HLA class I protein may have an important prognostic value for tumor patients. However, before now there was no consistent conclusion regarding the role of HLA class I molecules in cancer development for survival time. To the best of our knowledge, this is the first meta-analysis examining the prognostic value of HLA class I overexpression in gastrointestinal cancers.

Our meta-analysis incorporated 10 eligible studies. Patient survival outcomes were evaluated using hazard ratio (HR). Our results showed that HLA class I overexpression may have a significant positive association with overall survival (HR: 0.72; 95% CI (0.53, 0.96)). But for recurrence- free survival, overexpression probably has little impact on survival (HR: 0.70; 95% CI: (0.46, 1.08)).

Importantly, results of pooled OR concerning tumor differentiation and also tumor stage, revealed that the chance of overexpression in the advanced grades is less than the lower grades and overexpression tends to be found in the lower stages. In the previous studies the role of tumor differentiation has been proven several times. The Panzuto *et al*., study revealed that for gastro- entero-pancreatic endocrine tumors the poor degree of differentiation were identified as prognostic factor with a negative effect on survival time^41^. About tumor stage, researches confirmed that the higher disease stage, results in the lower patient survival. In a study by NG *et al*., on gastrointestinal leiomyosarcomas, it has been shown that 5 years overall survival in patients with advanced stage is significantly less than the other stages^42^. In the present study the pooled OR findings were consistent with the mentioned study and also emphasize our results on patient survival. The authors explain due to incompleteness of distal metastasis information in the extracted studies, they did not use this variable in the pooled analysis.

In the present research, heterogeneity was observed among the results of the included studies. It is necessary to point that because of few numbers of extracted studies for meta- analysis, the author did not perform meta- regression however, we tried to find the potential cause of heterogeneity through subgroups. Subgroup results had little benefit for this goal and maybe the reason was few number of study in each category. Variety in the methods for HLA- I expression finding, definition of immunohistochemical staining of specimens and using different antibodies are the factors which may have influence on the heterogeneity. It is believed that there is a need for precise identification of HLA class I expression on tumor cells, because of its important role in tumor cell recognition by CTL and NK cells. Nevertheless, this process shows technical difficulties and problems with the result interpretation in solid tumors.

In addition, publication bias also was found in the meta- analysis. Publication bias tends to be remarkable if there is unpublished studies with smaller sample size and if there is studies with suspicious and unreliable result^43^. The authors performed trim and fill analysis to adjust this problem. This analysis reinforced the significant prognostic role of HLA- I on overall survival of gastrointestinal cancer^44^. Moreover, the authors tried to cover most of databases as possible in the study extraction process but the limitation of English published study, may effects the publication bias.

This point that the HR and CI were calculated indirectly from some studies, may makes bias in the estimates and leads to a cautious interpretation of our results. The studies with large sample size, more accurate and complete results and also studies by using standard method and finally uniformed system for scoring, makes more confident result in the prognostic role of HLA- I for gastrointestinal cancers. Such studies are suggested for future researches.

Although our research only studied the behavior of HLA-A, B, and C in gastrointestinal cancers, it is important to examine the role of non- classical HLA (HLA-G). In a study, Murdaca *et al*. investigated the prognostic role of HLA-G in gastric adenocarcinoma patients. The findings of their study suggest that HLA-G overexpression is associated with poor survival in patients with stage III^45^. In addition to the prognostic role of HLA-G, also its serum level behavior is important in some diseases, including Human Immunodeficiency Virus (HIV) and chronic Hepatitis C Virus (HCV). HLA-G can be considered as a useful biomarker for monitoring viral responses and immune remodeling in patients with these diseases. Previous studies have shown that serum levels of HLA-G decrease in HIV patients treated with Active Antiretroviral therapy (HAART), as well as in HCV patients treated with Ribavirin, by time^46,47^. The behavior of HLA-G in a variety of diseases may vary, and study of this topic may introduce a new way of treating patients.

## Conclusion

This meta-analysis of prospective cohort studies demonstrated that HLA class I overexpression may be helpful marker for the clinical decision-making process regarding gastrointestinal cancer treatment and outcomes. Further studies using additional putative prognostic markers in combination with HLA class I may be required to evaluate their potential in predicting patient outcomes.
